# Effect of microclimate alteration on milk production and composition in Murrah buffaloes

**DOI:** 10.14202/vetworld.2015.1444-1452

**Published:** 2015-12-29

**Authors:** Sandeep Reddy Seerapu, Ananda Rao Kancharana, Venkata Seshaiah Chappidi, Eswara Rao Bandi

**Affiliations:** 1 Department of Livestock Production Management, NTR College of Veterinary Science, Sri Venkatesawara Veterinary University, Tirupathi, Andhra Pradesh, India; 2 Buffalo Research Station VR Gudem, Sri Venkateswara Veterinary University, Tirupathi, Andhra Pradesh, India

**Keywords:** microlimate, milk composition, milk yield, physiological parameters, temperature-humidity index

## Abstract

**Aim:**

The aim of this study was to assess the effect of microclimate alteration on temperature-humidity index (THI), milk yield, and milk composition of Murrah buffaloes during summer for a period of 90-day from March to May-2014 at Buffalo Research Station, Venkataramannagudem, Andhra Pradesh, India.

**Materials and Methods:**

A total of 40 lactating Murrah buffaloes were selected having similar body weight, parity, and milk yield. They were divided into four groups of 10 each. Three groups of buffaloes were provided with microclimate alteration using supplemental cooling like foggers, fans and foggers plus fans, and the fourth group (control) was without any cooling system. The daily THI was measured using dry and wet bulb thermometer. The physiological responses *viz*. rectal temperature, respiration rate, and pulse rate were measured by a clinical thermometer, measuring the flank movements a minute and observing the pulsation of the middle coccygeal artery at the base of tail with the help of finger. Milk samples were analyzed for chemical composition *viz*., fat, solids-not-fat (SNF), total solids (TS), specific gravity.

**Results:**

In the present study, significant (p<0.001) decrease in the average THI values were observed in experimental Murrah buffalo houses of GroupII (foggers), GroupIII (fans), and GroupIV (foggers and fans) compared to GroupI (control). Significant (p<0.001) decrease in average rectal temperature (°F), respiration rate (breaths/min) and pulse rate (beats/min) values were recorded in Murrah buffaloes of Groups II, III and IV compared to Group I. Significant (p<0.001) increase in the average milk yield (kg/day) was recorded in Murrah buffaloes of Groups II, III, and IV compared with Group I. Significant (p<0.001) increase in the average milk fat, SNF, and TS percent were recorded in Murrah buffalo Groups of II, III, and IV compared with Group I.

**Conclusion:**

Microclimate alteration by the provision of foggers and air circulators in the buffalo houses increased feed intake in buffaloes resulting increased milk production, fat and SNF yield which was due to decreased heat stress in buffaloes.

## Introduction

Buffalo is the second significant contributor to milk, meat, leather, and fuel. India possesses worlds’ highest population of buffaloes [1]. One of the greatest challenges being faced by producers and livestock around the world is thermal stress. Thermal stress is a major limiting factor in livestock production under tropical climate and also during the summer season in temperate climates. Thermal stress strongly affects animal bioenergetics, with adverse effects on the performance and well-being of livestock. Heat stress can have a very negative impact on milk production, reproduction and general health of livestock [2-4].

Meteorological variables which influence the ambient temperature significantly are dry bulb temperature, wet bulb temperature, wind velocity and intensity of solar radiation and radiations from surrounding structures in the animal byre. In tropical and subtropical areas, the high ambient temperature is the major constraint on animal productivity, and the effect of heat stress is aggravated when heat stress is accompanied with high humidity [5]. The heat stress affect the physiological systems governing thermal regulation and the maintenance energy of buffalo during extreme summer. Temperature-humidity index (THI) is the most common and most accurate mean of temperature stress assessment [6] and will be used to determine the influence of heat stress on productivity of dairy animals.

Exposure of buffaloes to hot climatic conditions leads to decreased production potential. Nutrient intake of high-producing buffaloes is closely related to the amount of milk production. The metabolizing nutrients generate heat, which may contribute to body temperature maintenance in a cold environment. However, in a warm climate, this heat needs to be dissipated to maintain body temperature and normal physiological functions. This complex interplay between physical and environmental effects influences the physiological functions of lactating animals and affects not only their milk production but also the efficiency of production, in turn, buffalo profitability.

Provision of supplementary cooling system like fans and foggers can reduce the effects of heat stress on dairy buffaloes in hot and humid climate of Coastal Andhra Pradesh of India. Use of water as a cooling agent is an effective method for reducing heat stress of livestock particularly under high ambient temperature and to keep their body temperature as cool as possible. The sprinkler system combined with forced air movement help increase the loss of body heat up to three- or four-folds [7]. Foggers disperse very fine droplets of water which quickly evaporate and cool the surrounding air. If air is flowing over the animal with a velocity between 2 and 3 m/s, it increases convective heat loss during stressful conditions; which can be achieved with air circulators [8]. Use of mist system for cooling cows and buffaloes was found to be economical also [9].

Therefore, the present work was envisaged to evaluate the effects of foggers and fans on physiological parameters and milk production and composition of Murrah buffaloes. Thus, the results would be helpful in maintaining the production efficiency of Murrah buffaloes during summer with successful implementation of evaporative cooling measures at the field level.

## Materials and Methods

### Ethical approval

The experiment was conducted after taking necessary permission by the Institutional Ethics Committee of Buffalo Research Station, Venkataramannagudem, Sri Venkateswara Veterinary University, Andhra Pradesh.

### Experimental design

The present study was conducted at Buffalo Research Station, Venkataramanagudem, West Godavari district of Andhra Pradesh. The institute was located at the upland area where the water source is mainly dependent on rainfall and bore well. The institute is situated at 16.49’ N latitude and 81.30’ E. The average annual rainfall is around 750 mm. The average temperature ranges from 25 to 45°C. The experiment was conducted for 90 days during the months of March, April and May of 2014 (hot-humid season). 40 lactating Murrah buffaloes were selected having similar body weight, parity and milk yield (completely randomized design). They were divided into four groups of 10 each. Details of the experimental buffaloes were presented in Tables-1-4. The buffaloes in Group I were kept without foggers and fans (control) while Group II buffaloes were provided with foggers, while Group III buffaloes were provided with fans and Group IV buffaloes were provided with both foggers and fans from 11.00 A.M. to 4.00 P.M. daily. Plastic foggers (BLUESTAL Agri equipment) with four way anti-leak technology with nozzle size 0.5mm and droplet size-80-100 µ are used in experimental Murrah buffalo houses. The experimental buffaloes were housed under loose housing system (asbestos roof over mangers). All the experimental buffaloes were offered 20kg chopped hybrid Napier and *ad-libitum* chopped maize straw as roughage source to meet their dry matter requirements. Concentrate feed was given at the time of milking as per the milk production of buffaloes (1kg concentrate per 2kg of milk).

**Table-1:** Details of experimental buffaloes: Group I (control).

Animal no	Body weight	Parity	Stage of lactation	Milk yield (in kg)
M-230	413	1	69	6.6
M-233	419	1	78	6.9
M-282	428	1	83	6.4
M-755	432	2	79	7.1
M-274	452	3	89	7.0
M-604	450	3	100	6.9
M-602	438	3	96	7.1
M-2661	455	4	102	7.3
M-558	450	4	92	7.0
M-463	460	5	107	7.1
Mean±SE	439.7±5.1	2.7±0.45	89.50±3.8	6.94±0.08

SE=Standard error

**Table-2:** Details of experimental buffaloes: Group II (foggers).

Animal no	Body weight	Parity	Stage of lactation	Milk yield (in kg)
M-296	400	1	82	6.5
M-832	425	1	110	7.0
M-847	410	1	91	6.8
M-236	430	2	112	7.3
M-761	435	3	99	7.1
M-408	447	3	97	7.0
M-403	429	3	79	6.6
M-2669	459	4	68	6.8
M-476	442	4	93	7.2
M-405	463	5	82	7.0
Mean±SE	434.0±6.2	2.7±0.45	91.30±4.4	6.93±0.08

SE=Standard error

**Table-3:** Details of experimental buffaloes: Group III (fans).

Animal no	Body weight	Parity	Stage of lactation	Milk yield (in kg)
M-299	410	1	70	6.9
M-836	428	1	98	6.6
M-521	421	2	89	6.9
M-732	404	2	101	7.3
M-461	430	3	111	7.4
M-970	439	3	66	7.1
M-1022	460	3	90	7.0
M-2665	450	4	103	7.0
M-2653	455	4	116	6.9
M-2673	465	5	88	7.0
Mean±SE	436.2±6.7	2.8±0.42	93.20±5.1	7.01±0.07

SE=Standard error

**Table-4:** Details of experimental buffaloes: Group IV (foggers and fans).

Animal no	Body weight	Parity	Stage of lactation	Milk yield (in kgs)
M725	405	1	119	6.1
M342	415	1	69	7.0
M282	430	2	80	6.8
M798	424	2	101	6.9
M795	439	2	105	7.8
M156	440	3	89	6.9
M478	455	3	81	6.9
M2671	445	4	102	7.9
M2652	459	4	86	6.3
M410	445	5	107	7.4
Mean±SE	435.7±5.4	2.7±0.42	93.90±4.8	7.00±0.18

SE=Standard error

### THI

THI was calculated by using the following formula:

THI = db°F−{(0.55−0.55 RH) (db°F−58)} [10].

Where, T_db_: Dry bulb temperature (°F) and RH: Relative humidity (RH%)/100.

Dry bulb temperature was recorded using dry bulb thermometer and wet bulb temperature from a wet bulb thermometer (Zeal, UK). RH was calculated from dry bulb and wet bulb temperatures using Psychrometric chart (Indian Meteorological Department). THI was calculated thrice per day, and the average value of the three readings was taken as THI of the day. The physiological responses *viz*. rectal temperature, respiration rate, and pulse rate were measured by a clinical thermometer, measuring the flank movements per minute and observing the pulsation of the middle coccygeal artery at the base of tail with the help of finger respectively, at 8.00 A.M. in the morning for all the experimental buffaloes before providing fogger cooling and fans. Thereafter, observations were recorded at 4.00 P.M. from the experimental buffaloes after provision of cooling through foggers and fans.

### Milk production and composition

During the experimental period, milk yield of individual buffaloes was recorded daily morning and evening from all the four groups of buffaloes. Milk samples were collected from buffaloes of the four groups during the experiment daily for 90-day period. The aliquots of milk of A.M. and P.M. milking were pooled. Milk samples were analyzed for chemical composition *viz*., fat, solids-not-fat (SNF), total solids (TS), specific gravity. The milk samples were analyzed by Everest Eko Milk Analyzer Ultra Pro, at Buffalo research station. The analyzer gave the result of lactometer reading and fat percent.

The temperatures of the collected milk samples were noted at the time of analysis and corrected lactometer reading was obtained by using correction factor. Corrected lactometer reading = LR + CF.

Correction factor (+) = 0.1 × difference in temperature above 60°F

Correction factor (−) = 0.1 × difference in temperature below 60°F

SNF:

SNF% = 0.25CLR + 0.22F + 0.72 [11]

CLR = Corrected lactometer reading, F= Fat content in milk

TS:

TS% = 0.25CLR + 1.22F + 0.72 [11]

CLR = Corrected lactometer reading, F= Fat content in milk.

### Statistical analysis

Statistical analyses were performed using the SPSS IBM, version 22.0, SPSS, Chicago (US). Mean ± standard error were calculated as per Snedecor and Cochran, 1994.

## Results and Discussion

The environmental parameters *viz*. dry and wet bulb temperature (°F), RH% recorded during the study period of 90-day were used for calculation of THI, and the data is depicted in Table-5. The peak THI was reached at the 12th week of the experiment during May in all the experimental buffalo sheds. Significant (p<0.001) difference in THI values were observed in Groups II, III, and IV compared with Group I. This might be due to decrease in THI due to cooling effect caused by the provision of foggers, fans, foggers and fans for the Groups II, III and IV, respectively. The reports of decreasing THI values from 84.1 to 82.78 in Murrah buffalo houses [12] and 81-77 in cattle houses [13] due to mister cooling corroborated the present results.

**Table-5:** Effect of microclimate alterations on THI of Murrah buffalo houses during summer (mean±SE).

Period of experiment (weekly interval)	Group I (control)	Group II (foggers)	Group III (air circulators)	Group IV (foggers and air circulators)
1^st^	75.57±0.16	72.49±0.21	73.57±0.16	72.39±0.21
2^nd^	75.84±0.16	72.72±0.14	72.87±0.13	72.53±0.14
3^rd^	75.89±0.10	72.84±0.12	73.28±0.18	72.68±0.12
4^th^	75.88±0.14	73.47±0.10	74.89±0.23	73.33±0.10
5^th^	75.87±0.16	73.44±0.10	73.97±0.08	73.35±0.08
6^th^	75.94±0.29	73.38±0.08	74.19±0.08	73.30±0.07
7^th^	77.05±0.10	73.86±0.08	74.38±0.09	73.78±0.07
8^th^	78.60±0.30	74.07±0.09	75.13±0.09	73.99±0.08
9^th^	78.41±0.10	74.21±0.18	75.83±0.22	74.12±0.18
10^th^	78.87±0.25	74.91±0.08	76.40±0.10	74.84±0.08
11^th^	79.64±0.16	75.09±0.13	76.35±0.17	75.05±0.13
12^th^	78.68±0.13	75.24±0.19	76.61±0.14	75.34±0.14
13^th^	78.69±0.16	74.32±0.14	76.58±0.10	74.23±0.05
Mean±SE	77.30±0.16^c^	73.85±0.10^a^	74.93±0.14^b^	73.76±0.10^a^

a,b,c Values with different superscripts in a row differ significantly (p<0.001). THI=Temperature-humidity indices, SE=Standard error

Significant (p<0.001) difference in THI was also observed in II and IV groups of buffalo houses compared to Group III buffalo house. Since all the experimental buffaloes are housed in loose houses, an evaporative cooling effect caused by foggers in II and IV buffalo houses might lower the THI values compared to III buffalo house which was equipped with fans alone. Dairy cattle barns equipped with fans and fans plus misting recorded temperatures of 31.5°C and 30.9°C, respectively [14] supporting the findings of effectiveness of the evaporative cooling system in decreasing the temperature in cattle houses over air circulation by fans alone.

However, the difference in THI between II and IV groups of buffalo houses were not significant due to free circulation of air in loose houses which aids evaporative heat loss without fans and THI was reduced on par with Group IV. Similar findings of the non-significant difference of THI were reported in cattle houses e quipped with universal oscillating fogger system and misting with Schaffer fan system, respectively [15].

The peak THI in Groups I, II, III, and IV was reached at the 12^th^ week of the experiment during May month in all the experimental buffalo houses. This might be due to increased atmospheric temperatures from March to May month during the experimental period. Upadhyay *et al*. [16] reported increased THI from February onward and exceeded 75 from March onward and in April ranged between 81 and 85 and THI exceeded 85 (86-95) during May in Murrah buffalo houses in India.

### Physiological parameters

The effect of microclimate alteration on mean rectal temperature (°F), respiration rate/min and pulse rate/min are presented in Tables-6-8.

**Table-6:** Effect of microclimate alteration on rectal temperature (°F) in Murrah buffaloes during summer.

Period of experiment (15-days interval)	Group I (control)	Group II (foggers)	Group III (fans)	Group IV (foggers and fans)
15	101.7±0.10	101.5±0.08	101.4±0.08	101.5±0.04
30	102.0±0.10	101.6±0.04	101.4±0.06	101.5±0.04
45	102.5±0.03	101.6±0.05	102.3±0.07	101.6±0.04
60	102.7±0.09	101.5±0.05	102.4±0.07	101.4±0.04
75	103.1±0.03	101.6±0.02	102.6±0.07	101.5±0.02
90	103.2±0.04	101.7±0.01	102.8±0.10	101.6±0.02
Mean±SE	102.5±0.06^c^	101.6±0.02^a^	102.1±0.06^b^	101.5±0.02^a^

a,b,c Values with different superscripts in a row differ significantly (p<0.001). SE=Standard error

**Table-7:** Effect of microclimate alteration on respiratory rate (breaths/min) in Murrah buffaloes during summer.

Period of experiment (15-days interval)	Group I (control)	Group II (foggers)	Group III (fans)	Group IV (foggers and fans)
15	32.17±0.54	19.83±0.31	23.41±0.26	20.50±0.26
30	34.63±0.52	21.32±0.36	23.02±0.22	21.88±0.29
45	39.28±0.46	21.65±0.34	24.28±0.70	22.01±0.27
60	39.73±0.28	22.92±0.31	30.93±0.95	23.29±0.24
75	40.42±0.16	23.18±0.38	33.16±0.33	23.04±0.33
90	40.64±0.15	24.02±0.90	35.14±0.51	24.29±0.88
Mean±SE	37.81±0.37^c^	22.15±0.26^a^	28.32±0.58^b^	22.50±0.23^a^

a,b,c Values with different superscripts in a row differ significantly (p<0.001). SE=Standard error

**Table-8:** Effect of microclimate alteration on pulse rate (beats/min) in Murrah buffaloes during summer.

Period of experiment (15-days interval)	Group I (control)	Group II (foggers)	Group III (fans)	Group IV (foggers and fans)
15	63.16±0.45	46.74±0.27	53.53±0.28	47.72±0.25
30	64.47±0.39	48.92±0.35	54.72±0.30	50.37±0.24
45	66.16±0.37	51.90±0.31	54.66±0.35	53.01±0.33
60	68.77±0.67	53.77±0.51	56.69±0.30	53.25±0.40
75	72.21±0.26	53.26±0.31	60.08±0.71	53.37±0.22
90	72.38±0.29	53.77±0.38	63.07±0.39	54.28±0.22
Mean±SE	67.86±0.41^c^	51.39±0.32^a^	57.12±0.40^b^	52.00±0.26^a^

a,b,c Values with different superscripts in a row differ significantly (p<0.001). SE=Standard error

Significantly (p<0.001) lower mean rectal temperature (°F), respiration rate/min and pulse rate/min was observed in II, III, and IV groups of buffaloes compared to Group I. This might be due to decrease in heat stress due to cooling effect caused by provision of foggers, fans, foggers and fans for the Groups II, III, and IV, respectively. Similar findings of decreased rectal temperature of 102.2°F was reported by Aggarwal [12] in dairy cows kept in houses equipped with mister cooling compared to without mister cooling system (103.84°C). Similar rectal temperature values of 38.5°C and 39.3°C in cows [17] and 38.7°C and 39.4°C in crossbred cows [18] and 38.5±0.3°C and 39.3±0.3°C in dairy cattle [19] provided with and without supplemental cooling system, respectively were consistent with the present results.

Respiration rate was the important physiological response that is clearly evident and increased with the heat stress. Decreased respiration rate due to the provision of foggers reported in dairy cows [17,18] and buffaloes [12,20] were corroborated with the present findings.

Pulse rate is a stress indicator which can be altered with thermal stress. Decreased pulse rate (68.7/min) in buffaloes kept under showers compared to buffaloes without showers (87.8/min) was reported by Aggarwal and Singh [21].

Significant (p<0.001) difference in mean rectal temperature (°F), respiration rate/min and pulse rate/min was also observed in II and IV groups of buffaloes compared to Group III might be due to evaporative cooling effect caused by foggers in experimental buffaloes housed in Group II and IV compared to Group III. Rectal temperatures of 38.64°C and 39.49°C in cattle [14] and 99.98 and 101.39°F in buffaloes [22] equipped with fans plus misting and fans, respectively corroborated the present findings. Similar results of respiration rate and pulse rates of 29.38 and 37.82 and 57.82 and 68.12/min, respectively in Nili-Ravi buffaloes reported by Das *et al*. [22].

However, the difference in mean rectal temperature (°F), respiration rate/min and pulse rate/min between II and IV groups of buffaloes were not significant. Even though the fans were not provided in Group II buffalo house, evaporative cooling had taken place due to free circulation of air and the rectal temperature of Group II buffaloes were comparable with Group IV buffaloes. Non-significant difference in rectal temperatures of 39.4±0.07°C and 39.6±0.04°C in dairy cows housed in universal oscillating fogger system and misting with Schaffer fan system, respectively, reported by Noniponimo [15] corroborated with the present findings. Similarly, non-significant difference in respiration rate of 28.78 and 28.49 counts/min in splashing and wallowing groups of Murrah buffaloes [23] and pulse rate of 77.10 and 78.58/min in crossbred cattle [24] were reported.

The highest mean rectal temperature (°F), respiration rate/min and pulse rate/min in experimental buffaloes of Groups I, II, III, and IV, respectively was reached during last week of May of the experiment. This was due to increased ambient temperatures from March to May during the experimental period which might be responsible for the increase in rectal temperature of the experimental buffaloes. Koubkova *et al*.[25] reported the rectal temperature of 39.3°C in Holstein cows exposed to high ambient temperature conditions during peak summer. Increased rectal temperature in dairy cows during peak summer corroborated the present findings [26-28]. Higher respiration rate of 71.5/min during summer compared to 38.8/min in winter in lactating cows [26] and pulse rate of 56.50±0.63 and 64.50±0.42 in adult Sahiwal cattle [13] in spring and summer seasons, respectively, were consistent with present findings.

### Milk production

The effect of microclimate alteration in the experimental buffalo houses on milk production (kg) and milk constituents (fat, SNF and TS) in buffaloes are presented in Tables-9-12. The mean milk production (kg) in experimental buffaloes was 6.05± 0.02, 7.30±0.01, 6.75±0.01, and 7.31±0.01 kg/day, respectively, in Groups I, II, III, and IV.

**Table-9:** Effect of microclimate alteration on milk yield (kg) in Murrah buffaloes during summer.

Period of experiment (15-days interval)	Group I (control)	Group II (foggers)	Group III (fans)	Group IV (foggers and fans)
15	6.28±0.03	7.30±0.02	6.77±0.02	7.31±0.01
30	6.17±0.02	7.31±0.02	6.77±0.01	7.31±0.02
45	6.09±0.01	7.30±0.02	6.75±0.02	7.33±0.02
60	6.00±0.02	7.30±0.01	6.72±0.02	7.31±0.02
75	5.87±0.01	7.31±0.02	6.76±0.02	7.33±0.01
90	5.86±0.01	7.30±0.01	6.72±0.02	7.30±0.01
Mean±SE	6.05±0.02^c^	7.30±0.01^a^	6.75±0.01^b^	7.31±0.01^a^

a,b,c Values with different superscripts in a row differ significantly (p<0.001). SE=Standard error

**Table-10:** 

Period of experiment (15-days interval)	Group I (control)	Group II (foggers)	Group III (fans)	Group IV (foggers and fans)
15	6.06±0.04	7.09±0.01	6.64±0.02	7.07±0.02
30	6.00±0.01	7.08±0.02	6.56±0.03	7.05±0.01
45	6.00±0.02	7.07±0.02	6.50±0.04	7.04±0.01
60	5.98±0.01	7.09±0.02	6.61±0.02	7.05±0.01
75	5.94±0.01	7.07±0.02	6.60±0.02	7.04±0.02
90	5.93±0.01	7.02±0.03	6.43±0.03	7.03±0.02
Mean±SE	5.99±0.01^c^	7.07±0.01^a^	6.56±0.01^b^	7.05±0.01^a^

a,b,c Values with different superscripts in a row differ significantly (p<0.001). SE=Standard error

**Table-11:** Effect of microclimate alteration on milk SNF (%) in Murrah buffaloes during summer.

Period of experiment (15-days interval)	Group I (control)	Group II (foggers)	Group III (fans)	Group IV (foggers and fans)
15	9.14±0.02	10.07±0.02	9.42±0.02	10.08±0.02
30	9.17±0.03	10.05±0.02	9.47±0.02	10.09±0.02
45	9.16±0.03	10.05±0.02	9.41±0.02	10.07±0.02
60	9.16±0.03	10.01±0.02	9.36±0.05	10.05±0.02
75	9.12±0.02	9.99±0.03	9.36±0.03	10.01±0.02
90	9.11±0.02	9.99±0.02	9.21±0.04	10.01±0.02
Mean±SE	9.14±0.01^c^	10.03±0.01^a^	9.37±0.02^b^	10.05±0.01^a^

a,b,c Values with different superscripts in a row differ significantly (p<0.001). SNF=Solids-not fat, SE=Standard error

**Table-12:** Effect of microclimate alteration on milk TS (%) in Murrah buffaloes during summer.

Period of experiment (15-days interval)	Group I (control)	Group II (foggers)	Group III (fans)	Group IV (foggers and fans)
15	15.20±0.05	17.14±0.03	16.06±0.03	17.15±0.03
30	15.17±0.03	17.13±0.03	16.06±0.03	17.12±0.03
45	15.16±0.05	17.12±0.04	16.02±0.03	17.11±0.03
60	15.14±0.03	17.10±0.04	15.92±0.06	17.07±0.03
75	15.07±0.03	17.07±0.03	15.87±0.09	17.08±0.03
90	15.03±0.02	17.01±0.06	15.64±0.07	17.06±0.03
Mean±SE	15.13±0.01^c^	17.10±0.02^a^	15.93±0.03^b^	17.10±0.01^a^

a,b,c Values with different superscripts in a row differ significantly (p<0.001). SE=Standard error, TS=Total solids

Significantly (p<0.001) higher milk production was observed in II, III, and IV groups of buffaloes compared to Group I. This might be due to decrease in heat stress on buffaloes housed in houses equipped with foggers, fans and foggers plus fans in Groups II, III and IV, respectively, resulting increased feed and water intake and increased milk production compared to Group I. The effects of cooling on milk yield probably could be due to change in rectal temperature, diurnal rhythm [29] or by restoring feed intake is corroborating the present findings.

Similar results of increased milk production of 1.9 kg/day in dairy cows cooled by sprinklers and fans during summer [30] and 15% increase in milk yield in Friesian cows provided with ventilation and sprinkling during summer [31] corroborating the present findings. Aggarwal [12] reported that the milk production in Murrah buffaloes was 7.58 and 8.60 in control (allowed for wallowing) and mister system, respectively. Similarly, milk production of 16.9±1.9 and 12.6±0.6 kg/day in crossbred primiparous cows housed under evaporative cooling housing system and open housing system were reported by Chanpongsang *et al*. [18] and milk yield of 5.63 and 5.32 in Murrah buffaloes maintained under the high-pressure fogger system, and control were reported byAmbulkar *et al*. [20] which corroborate with the present findings.

Significant (p<0.001) difference in milk production was also observed in II and IV groups of buffaloes compared to Group III buffaloes. This might be due to evaporative cooling effect caused by foggers which further aids inadequate feed intake in the experimental buffaloes housed in Groups II and IV. Similarly, results of milk yield of 29.0 and 29.8kg/day were reported by Frazzi *et al*. [14] in dairy cattle barns that are equipped with fans and fans plus misting, respectively. Das *et al*. [22] reported the mean daily milk production was 6.9 kg and 8.1 kg/day in Nili-Ravi buffaloes supplemented with ceiling fans and foggers/misting fans.

However, the difference between II and IV groups of buffaloes were not significant. Even though the fans were not provided in Group II buffalo house evaporative cooling had taken place and due to free circulation of air and the milk yield values were comparable with Group IV. These findings are in accordance with Shiao *et al*. [32] who reported non-significant difference in milk production values of 25.0, 24.9 and 25.1 kg/day in three different cooling systems, a tunnel-ventilated and water-padded free stall barn, a conventional free stall barn with fans and sprinklers in the feed line and a TP barn with sprinkler cooling, respectively.

The lower milk production values of 5.86±0.01, 7.30±0.01, 6.72±0.02, and 7.30±0.01kg/day in the experimental buffaloes were reached at last week of May during the experiment. This was due to increased THI from March to May of the experimental period which was responsible for decreased feed intake which might directly affect the milk production in the experimental buffaloes. Barash *et al*. [33] found a depression in milk production in dairy cows subjected to heat stress and suggested that part of energy cost for milk production is diverted to maintain body temperature. Baknik *et al*. [34] reported a decrease of 0.18 kg/day milk yield in cows for 1°C rise in environmental temperature and Bouraoui *et al*. [35] reported daily milk yield decreased by 21% when THI exceeded 78 in cows during summer corroborating with present findings. Similarly decreased milk yield from 22 kg to about 18 kg in dairy cows at THI of 83 in summer [36] and a decrease of milk yield of 1.27 kg/day in Italian Holstein dairy cattle [37] at THI 76 were consistent with the present study.

The availability of showers/water sprinklers over the lactating animals reduces heat stress resulting the milk production can either be sustained [38] or enhanced [29,39]. Since higher basal values of glucose are required for synthesis of lactose, the improvement in milk production could probably be attributed to higher glucose in experimental animals because of more feed intake [40].

### Milk constituents

The milk constituent’s *viz*., milk fat, SNF, and TS were decreased to a minimum level in all the experimental groups during last week of May of the experiment, and the values are presented in Tables-6-8. The average milk fat percent was 5.99±0.01, 7.07±0.01, 6.56±0.01 and 7.05±0.01 for the Groups I, II, III and IV, respectively. The mean milk SNF in experimental buffaloes was 9.14±0.01, 10.03±0.01, 9.37±0.02 and 10.05±0.01%, respectively for the Groups I, II, III and IV and the mean milk TS per cent was 15.13±0.01, 17.10±0.02, 15.93±0.03, and 17.10±0.01for the Groups I, II, III and IV, respectively.

The milk fat, SNF and TS were significantly (p<0.001) increased in buffalo Groups II, III and IV compared with Group I. This might be due to decrease in heat stress on buffaloes caused by provision of foggers, fans and foggers plus fans in buffalo houses of Groups II, III and IV, respectively. Aggarwal [12] reported that the milk fat and milk TS were 6.92 and 7.42 and 17.41 and 18.23 in buffaloes housed without misters and misters, respectively is inconsistent with present findings.

Significant (p<0.001) difference in milk constituents was also observed in II and IV groups of buffaloes compared to Group III buffaloes. This might be due to evaporative cooling effect caused by foggers which further aids inadequate feed intake in the experimental buffaloes housed in Groups II and IV compared with Group III. Similar findings of increased milk fat in dairy cattle [41] housed under sprinklers (3.66±0.59%) compared to fans (3.61±0.53) corroborate with the present study.

However, the difference between II and IV groups of buffaloes were not significant. Even though the fans were not provided in Group II buffalo house, evaporative cooling had taken place due to free circulation of air which might be responsible for non-significant difference in the milk constituents between Groups II and IV. The results of the present study were in agreement with the findings of Shiao *et al*. [32], who reported 4.05, 4.06 and 4.02 fat % in three different cooling systems *viz*. tunnel-ventilated and water-padded free stall barn, a conventional frees tall barn with fans and sprinklers in the feed line and a TP barn with sprinkler cooling, respectively in Holstein dairy cattle.

The lower values of milk fat percent of 5.93±0.01, 7.02±0.03, 6.43±0.03, and 7.03±0.02 and SNF percent of 9.11±0.02, 9.99±0.02, 9.21±0.04, and 10.01±0.02 and TS percent of 15.03±0.02, 17.01±0.06, 15.64±0.07, and 17.06±0.03 in Group I, II, III, and IV, respectively. This might be due to increased heat stress in buffaloes from March to May of the experimental period which was responsible for decreased feed intake resulting decreased milk constituents all the experimental buffaloes. The results obtained in the present study were in accordance with the findings of Itoh *et al*.[40] who reported decrease in milk fat from 3.7% to 3.3% in dairy cows in summer. Lambertz and Gauly [42] who also reported increase in THI from 65 decreased the milk fat in Holstein cows.

## Conclusion

The most common way to alleviate heat stress of buffaloes and their environment is by evaporative cooling. This study provides information regarding effectiveness by the use of evaporative cooling system through foggers and fans is an efficient method for reduction of heat stress in buffalo sheds. The results also indicated that reduced physiological parameters mainly rectal temperature, respiration, and pulse led to increased feed intake and higher milk yield and milk composition in microclimate altered groups during summer. Therefore, supplementation of foggers for 5 h/day from 11.00 am to 4.00 pm can be recommended in tropical conditions under loose housing system to combat heat stress and improve the performance.

## Authors’ Contributions

SS: Recording of physiological and milk production parameters on buffaloes housed in different microclimate modified houses, analysis of milk constituents and preparation of manuscript. AK: Technical help in microclimate modification and feeding management of buffaloes. VC: Design of the experiment and THI calculation. ER: Data analysis. All authors read and approved the final manuscript.
